# Perforated Small Intestinal Diffuse Large B-Cell Lymphoma Successfully Managed With Surgical Resection and Staged Introduction of Polatuzumab Vedotin-Based Chemotherapy

**DOI:** 10.14740/jmc5262

**Published:** 2026-02-02

**Authors:** Shunsuke Yamada, Yoshikazu Ikoma, Tomohiro Aizaki, Kiichi Otani, Yuto Kaneda, Yuta Sato, Takuro Matsumoto, Nobuhiko Nakamura, Hiroshi Nakamura, Nobuhisa Matsuhashi, Nobuhiro Kanemura, Masahito Shimizu

**Affiliations:** a Department of Hematology and Infectious Disease, Gifu University Hospital, Gifu, Japan; b School of Medicine Medical Course, Gifu University, Gifu, Japan; c Center for Nutrition Support and Infection Control, Gifu University Hospital, Gifu, Japan; d Department of Hematology, Gifu Municipal Hospital, Gifu, Japan; e Department of Gastroenterology, Gifu University Hospital, Gifu, Japan; f Department of Gastroenterological Surgery and Pediatric Surgery, Gifu University Graduate School of Medicine, Gifu, Japan; g These authors contributed equally to this work.

**Keywords:** Diffuse large B-cell lymphoma, Gastrointestinal lymphoma, Polatuzumab vedotin, Surgical procedures, Intestinal perforation

## Abstract

Polatuzumab vedotin in combination with rituximab, cyclophosphamide, doxorubicin, and prednisone (Pola-R-CHP) is a vincristine-free regimen recently approved for untreated diffuse large B-cell lymphoma (DLBCL). However, its safety in the early postoperative setting remains unclear. We report a case of primary intestinal DLBCL with suspected perforation managed with surgical resection followed by staged introduction of Pola-R-CHP. Rituximab was started on postoperative day 11, followed by CHP on day 15 as the first cycle of R-CHP. Pola was added from cycle 2. The patient showed an initial tumor reduction without serious gastrointestinal (GI) toxicity. This case suggests that staged introduction of Pola-R-CHP may minimize postoperative GI risk while maintaining safety.

## Introduction

Diffuse large B-cell lymphoma (DLBCL) frequently affects extranodal sites, and gastrointestinal (GI) involvement occurs in approximately 30–40% of extranodal lymphomas and 4–20% of all non-Hodgkin lymphomas [1]. Among GI lymphomas, primary intestinal DLBCL is clinically significant because of its high risk of serious complications, such as perforation, bleeding, and obstruction [2]. Approximately 13% of patients with DLBCL involving the GI tract experience perforation, which can significantly compromise clinical outcomes [2]. To prevent these complications, early surgical resection prior to chemotherapy has been proposed as a viable approach, supported by recent evidence demonstrating improved survival outcomes with the combination of surgery and chemotherapy compared with chemotherapy alone [3, 4].

For many years, the standard first-line therapy for DLBCL has been rituximab combined with cyclophosphamide, doxorubicin, vincristine, and prednisone (R-CHOP). Recently, in the pivotal phase III POLARIX trial, polatuzumab vedotin (Pola), an antibody–drug conjugate targeting CD79b, was combined with rituximab, cyclophosphamide, doxorubicin, and prednisone, resulting in a vincristine-free regimen termed Pola-R-CHP [5]. This regimen demonstrated superior efficacy compared with R-CHOP. In that trial, the most common GI adverse events associated with Pola-R-CHP were diarrhea (31%), nausea (42%), and constipation (29%), which were primarily grade 1–2 and generally manageable with supportive care. Although rare, serious GI complications such as gastric perforation have also been documented in isolated reports involving Pola-containing regimens [6]. However, the safety and optimal timing of Pola-R-CHP therapy immediately following GI surgery remain unclear. Specifically, it remains unclear whether Pola can be safely administered soon after surgical intervention for intestinal DLBCL, or if a staged introduction of Pola might mitigate GI risks more effectively.

To answer this clinical question, we present a case of a primary small intestinal DLBCL complicated by perforation. This patient underwent initial surgical resection followed by staged Pola-R-CHP chemotherapy initiated 2 weeks postoperatively. Through this case, we aim to clarify the safety and potential clinical benefit of introducing Pola-R-CHP in a staged manner following intestinal surgery.

## Case Report

**Figure 1:**
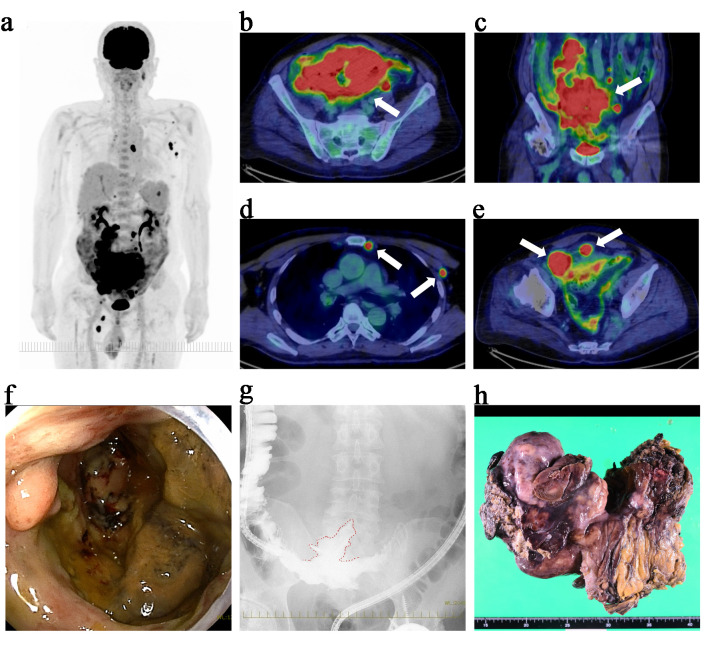
Initial findings prior to chemotherapy: PET–CT, endoscopy, contrast study, and surgical specimen. (a) Maximum intensity projection image showing extensive FDG uptake throughout the abdomen and pelvis. (b) Axial PET–CT view revealing a large hypermetabolic mass in the terminal ileum (white arrow) with a maximum standardized uptake value (SUV_max_) of 27.0. (c) Coronal PET–CT view demonstrating the same mass, confirming its extensive distribution in the pelvis (white arrow). (d) FDG-avid lymphadenopathy in the internal thoracic and left axillary regions (white arrows). (e) Axial view showing FDG uptake in the pelvis, suggestive of peritoneal thickening or mesenteric involvement (white arrows). (f) Transanal enteroscopy showing an ulcerated mass at the terminal ileum. Biopsy from this site confirmed the diagnosis of diffuse large B-cell lymphoma, not otherwise specified (DLBCL, NOS). (g) Contrast enema revealing localized extraluminal leakage of contrast medium (outlined in red), indicative of a contained perforation into the mesentery. (h) Macroscopic appearance of the resected specimen. The tumor, measuring approximately 25 cm at its greatest dimension, involved the right hemicolon and a segment of the terminal ileum, forming a single confluent mass. It was resected *en bloc*, and partial necrosis was observed on macroscopic examination. The scale at the bottom of the image is in centimeters. PET–CT: positron emission tomography–computed tomography; FDG: fluorodeoxyglucose.

A 62-year-old man presented with persistent fever and abdominal pain. Physical examination revealed a palpable mass in the lower abdomen, with mild tenderness and no signs of peritoneal irritation. Laboratory tests showed elevated lactate dehydrogenase (LDH, 353 U/L), C-reactive protein (4.43 mg/dL), and soluble interleukin-2 receptor (1,207 U/mL). Positron emission tomography–computed tomography (PET–CT) using fluorodeoxyglucose (FDG) revealed a large ileal mass with a maximum standardized uptake value of 27.0 (Fig. 1a–c). PET–CT also demonstrated FDG-avid lymphadenopathy in the mesenteric, bilateral internal thoracic, left axillary, and left supradiaphragmatic regions (Fig. 1d). In addition, FDG uptake in the pelvis was suggestive of peritoneal thickening or mesenteric involvement (Fig. 1e). Transanal enteroscopy revealed an ulcerated lesion at the terminal ileum (Fig. 1f). The subsequent contrast enema demonstrated localized extraluminal leakage, suggesting a contained perforation into the mesentery (Fig. 1g). A biopsy from the endoscopic lesion confirmed diffuse large B-cell lymphoma, not otherwise specified (DLBCL, NOS) [7]. Immunohistochemical analysis showed that the tumor cells were positive for CD20 and CD79a and negative for CD3, CD5, and CD10. BCL2 was weakly positive, BCL6 was focally positive, and MUM1 was positive, while MYC and cyclin D1 were negative. Epstein–Barr virus–encoded RNA *in situ* hybridization was negative. The Ki-67 (MIB-1) labeling index was approximately 90%. Based on the Hans algorithm, the lymphoma was classified as non-germinal center B-cell-like (non-GCB) type [8]. Fluorescence *in situ* hybridization (FISH) for MYC, BCL2, and BCL6 rearrangements was not performed. Staging evaluations indicated Ann Arbor stage IVB [9] and Lugano stage IV (1994 classification for GI lymphoma) [10], with an International Prognostic Index score of 4, thereby categorizing the patient as high risk [11].

**Figure 2:**
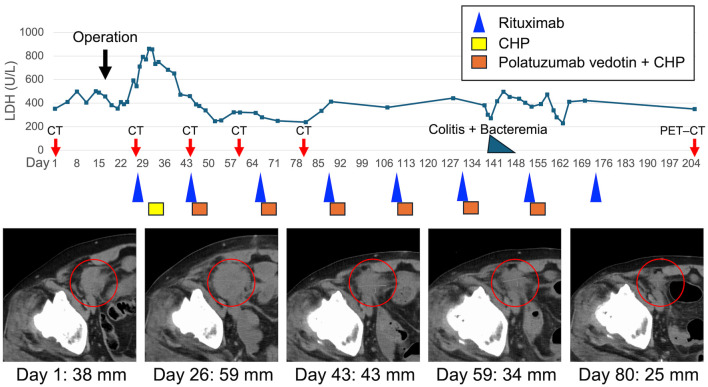
Clinical course with serum LDH kinetics, tumor response on CT imaging. LDH levels are plotted against days from initial presentation (day 1). The patient underwent right hemicolectomy with partial resection of the ileum on day 16. Chemotherapy was initiated with rituximab on day 27, followed by CHP on day 31. Polatuzumab vedotin was added from day 44 onward, completing the transition to the Pola-R-CHP regimen. CT images at days 1, 26, 43, 59, and 80 (red circles) demonstrate changes in the size of the pelvic tumor lesion over time, corresponding with the treatment response. Tumor diameters are indicated below each image. Colored boxes and symbols represent chemotherapy agents: rituximab (blue triangle), CHP (yellow box), and polatuzumab vedotin + CHP (orange box). The black arrow indicates the timing of surgery; red arrows indicate CT/PET–CT evaluation time points; and the blue-green triangle indicates the episode of colitis with bacteremia. CHP: cyclophosphamide, doxorubicin, and prednisone; CT: computed tomography; LDH: lactate dehydrogenase; PET–CT: positron emission tomography–computed tomography.

Due to intestinal perforation, surgical intervention was prioritized, and the patient underwent right hemicolectomy with partial resection of the ileum (Fig. 1h). Intraoperatively, a giant mass (approximately 25 cm in greatest dimension) was found occupying the pelvic cavity and forming an inflammatory conglomerate with the sigmoid colon and small bowel around an abscess cavity; however, the precise perforation site could not be clearly identified. The involved ileal segment (approximately 5 cm in length), which was severely involved and could not be preserved, was resected as part of an *en bloc* resection, and the abscess cavity was excised. Macroscopic complete resection of the intestinal mass (including the abscess cavity) was achieved; however, mesenteric lymphadenopathy remained. Although chemotherapy was initially planned to begin 1 month postoperatively, rapid disease progression and favorable postoperative recovery led to an earlier start. At the time of chemotherapy initiation, the patient demonstrated favorable postoperative recovery, including an Eastern Cooperative Oncology Group performance status of 1 [12], preserved renal and hepatic function, no signs of active infection, and had resumed oral intake without clinical evidence of significant malnutrition, which supported the decision to administer full-dose chemotherapy. Therefore, no dose reduction of R-CHP was considered necessary; however, Pola was intentionally withheld only in the first cycle to mitigate potential postoperative GI toxicity. Rituximab was administered on postoperative day 11, followed by CHP on day 15 as the first cycle of R-CHP. Because no GI events were observed with the first chemotherapy, Pola was added at a dose of 1.8 mg/kg from the second cycle onward, transitioning to the Pola-R-CHP regimen (Fig. 2).

During the fifth cycle, the patient developed colitis centered around the surgical site and *Bacteroides fragilis* bacteremia. These complications resolved promptly with antibiotic therapy without necessitating delays or modifications in the chemotherapy schedule. In total, the patient received six cycles of Pola and seven cycles of R-CHP. No grade 3 or higher adverse events were observed throughout the treatment course. CT scans during chemotherapy showed tumor shrinkage; however, post-treatment PET–CT scans revealed new lesions, indicating progressive disease. The patient currently remains on second-line chemotherapy.

## Discussion

Evidence regarding early postoperative initiation of Pola-R-CHP after intestinal perforation is limited; therefore, we report this case to share practical considerations for perioperative sequencing and safety. This case highlights two clinically relevant findings. First, full-dose Pola-R-CHP chemotherapy may be safely administered following surgical resection in patients with primary intestinal DLBCL. Second, a staged introduction of Pola—beginning with R-CHP alone and adding Pola from the second cycle onward—may represent a rational strategy to reduce postoperative GI risks while maintaining treatment intensity and schedule.

The first key observation is that Pola-R-CHP chemotherapy was safely implemented early in the postoperative period, without serious GI complications. Chemotherapy was initiated on postoperative day 14, which was earlier than initially planned because of rapid disease progression. Reported intervals between surgery and chemotherapy for intestinal lymphoma are typically around 2–6 weeks; for example, representative series report a median interval of 19.5 days (range, 14–42 days) and 29 postoperative days [13, 14]. Our initiation at postoperative day 14 was at the lower end of this range, and we withheld Pola only in cycle 1 to minimize potential early postoperative GI toxicity. The favorable course in this patient can be attributed to the surgical resection of the perforation-prone lesion, as well as sufficient recovery of the patient’s general condition. Although the optimal timing of chemotherapy following GI surgery remains individualized, the present case suggests that early resumption of systemic therapy may be feasible with careful patient selection. Importantly, the safety profile observed here is consistent with the phase III POLARIX trial [5], where Pola-R-CHP demonstrated non-inferior toxicity compared with R-CHOP, including a similar rate of GI adverse events such as diarrhea and nausea, with no significant increase in perforation or other critical GI events.

Second, this case supports the potential utility of a staged introduction of Pola in surgically treated intestinal DLBCL. Pola has been associated with GI side effects such as diarrhea [5], and while such events are generally manageable, concern exists regarding their impact in the early postoperative phase, particularly in patients with recent bowel anastomosis. In this context, we elected to omit Pola during the first chemotherapy cycle and introduce it from the second cycle onward after local healing was confirmed. Although no guidelines currently recommend this approach, it allowed us to mitigate early GI risks while maintaining the full treatment schedule. The patient completed six cycles of Pola and seven cycles of R-CHP without dose delays or grade ≥ 3 toxicity.

When considering the appropriateness of Pola-R-CHP in the postoperative setting, it is important to contrast it with R-CHOP, the previous standard regimen. Vincristine, a core component of R-CHOP, is associated with dose-limiting autonomic neurotoxicity, most notably paralytic ileus. This complication results from damage to the enteric nervous system, particularly the myenteric plexus, leading to bowel hypomotility and functional obstruction. While the incidence is relatively low, paralytic ileus can be life-threatening and has been reported even after a single dose, especially in postoperative or older patients [15]. Reports of R-CHOP-related GI complications in surgically treated DLBCL include both ileus and bowel perforation, often necessitating dose reductions or treatment delays [2, 3]. By contrast, rare but serious GI perforation has been reported with Pola-containing regimens [6]. However, Pola-R-CHP, which omits vincristine, has been associated with a lower incidence of neurotoxicity and GI-related adverse events compared with R-CHOP. In the POLARIX trial, grade ≥ 2 neuropathy occurred in 13.8% of patients receiving Pola-R-CHP versus 16.7% of those receiving R-CHOP, and discontinuation due to neurotoxicity was less frequent with Pola [5]. These data suggest that Pola-R-CHP may offer a safer alternative in patients at risk for GI dysfunction, such as those in the early postoperative setting, especially when compared with the known risk of vincristine-associated paralytic ileus.

In conclusion, this case offers two key insights: 1) Pola-R-CHP chemotherapy may be safely administered after bowel resection for primary intestinal DLBCL, and 2) a staged introduction of Pola is a practical and potentially safer approach in the early postoperative period. Although the disease unfortunately resulted in progressive disease despite completion of the planned treatment, this outcome is considered to reflect the limitations of the Pola-R-CHP regimen rather than a failure of the treatment strategy itself. Importantly, the strategy was feasible and well-tolerated, enabling early postoperative systemic therapy without serious GI complications and with an initial tumor reduction. Although the patient ultimately developed progressive disease, this experience highlights the importance of individualized treatment planning in patients with DLBCL accompanied by GI disease and supports consideration of Pola-based regimens as a potentially safe postoperative option.

This report has important limitations. Because it describes a single case, the safety, feasibility, and generalizability of early postoperative Pola-containing therapy cannot be established, and treatment efficacy cannot be inferred. Accordingly, this report should be regarded as hypothesis-generating, and further cases and studies are warranted to define optimal patient selection, timing, and sequencing.

### Conclusions

This case suggests that Pola-R-CHP chemotherapy may be safely administered following bowel resection for primary intestinal DLBCL. A staged introduction of Pola may represent a rational strategy to minimize postoperative GI risk. This approach was feasible and well-tolerated in the early postoperative setting and warrants further evaluation in surgically treated GI lymphomas.

## Data Availability

The data supporting the findings of this study are available from the corresponding author upon reasonable request.

## Abbreviations

DLBCL: diffuse large B-cell lymphoma
GI: gastrointestinal
R-CHOP: rituximab: cyclophosphamide, doxorubicin, vincristine, and prednisone
Pola: polatuzumab vedotin
Pola-R-CHP: Pola combined with rituximab, cyclophosphamide, doxorubicin, and prednisone
NOS: not otherwise specified
LDH: lactate dehydrogenase
PET–CT: positron emission tomography–computed tomography
